# Supplementary figures and images for: Exosomal miR-224 contributes to hemolymph microbiota homeostasis during bacterial infection in crustacean
Source: PLoS Pathog. 2021 Aug 11;17(8):e1009837. doi: 10.1371/journal.ppat.1009837 (PMC8382196; doi:10.1371/journal.ppat.1009837)

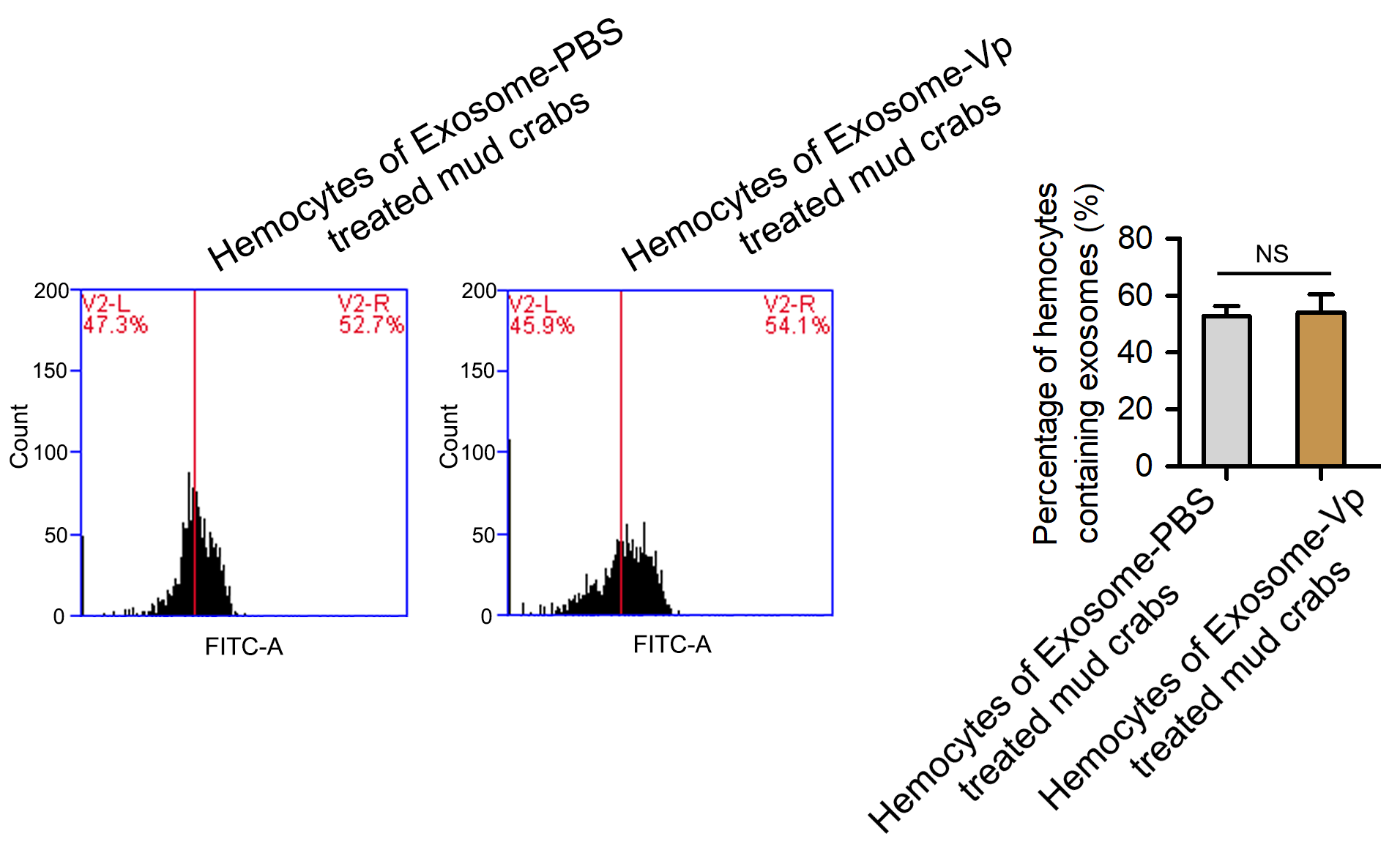

Supplement: S1 Fig — The indicated exosomes were labeled with Dio (green) for 6 h, after which the labeled exosomes were injected to the mud crab and then hemocytes were isolated and analyzed by flow cytometry. Experiments were performed in triplicates, with the data shown representing the mean ± s.d. (*, p<0.05; **, p<0.01). (TIF) [file ppat.1009837.s001.tif]
