# Supplementary material for: Exosomal miR-224 contributes to hemolymph microbiota homeostasis during bacterial infection in crustacean
Source: PLoS Pathog. 2021 Aug 11;17(8):e1009837. doi: 10.1371/journal.ppat.1009837 (PMC8382196; doi:10.1371/journal.ppat.1009837)
Supplement: S1 Table — (DOCX) [file ppat.1009837.s002.docx]

**S1 Table. LC-MS/MS data of IP products by HSP70 antibody**

| **Protein names** | **Score** |
| --- | --- |
| Transglutaminase  Integrin beta  Clotting protein  Heat shock protein 70  Hemocyanin subunit 1  Serine proteinase-like protein  Beta-actin  Ubiquitin  Hemocyanin subunit 2  Adaptor-related protein complex 2 beta 1 subunit (Fragment)  TNF receptor associated factor 6  Putative ras-related protein Rab (Fragment)  Beta-actin  Histone H2A  Heat shock protein family member  S3Ae ribosomal protein-like protein (Fragment)  Ras-related protein Rab-1A (Fragment)  Rab5 (Fragment)  Ribosomal protein L10 (Fragment)  Thioredoxin  Ubiquitin carboxyl-terminal hydrolase  Hypoxia inducible factor 1 alpha  Nitric oxide synthase  Macrophage migration inhibitory factor MIF1  Gamma-interferon induced thiol reductase GILT3  NADH-ubiquinone oxidoreductase chain 5  Myosin light chain  Replication factor C 2 (40kD) isoform 2 (Fragment) | 323.31  186.6  163.54  150.76 |
|  | 94.007  86.896  67.625  56.082  31.739  29.218  24.968  18.962  16.418  14.321  13.990  9.1452  6.7983  6.4271  4.3205  2.3572  2.2532  2.1855  1.8632  1.6527  1.2498  1.1835  -2  -2 |
