# Supplementary material for: Exosomal miR-224 contributes to hemolymph microbiota homeostasis during bacterial infection in crustacean
Source: PLoS Pathog. 2021 Aug 11;17(8):e1009837. doi: 10.1371/journal.ppat.1009837 (PMC8382196; doi:10.1371/journal.ppat.1009837)
Supplement: S2 Table — (DOCX) [file ppat.1009837.s003.docx]

**S2 Table. LC-MS/MS data of IP products by TRAF6 antibody**

| **Protein names** | **Score** |
| --- | --- |
| Glyceraldehyde-3-phosphate dehydrogenase  TNF receptor associated factor 6  Acidic p0 ribosomal protein (Fragment)  NADH-ubiquinone oxidoreductase chain 5  C-type lectine  Ras-related protein Rab-1A (Fragment)  Prophenoloxidase activating factor 2  Antimicrobial peptide hyastatin (Sphyastatin)  Ubiquitin  Minus strand heat shock protein 70  Beta-actin  Ecsit  Putative ras-related protein Rab (Fragment)  Ribosomal protein L10 (Fragment)  Hemocyanin subunit 3  Glyceraldehyde-3-phosphate dehydrogenase  Ribosomal protein L5 (Fragment)  Transglutaminase  Hemocyanin subunit 1  Histone H2A  PI3K (Fragment)  Replication factor C 2 (40kD) isoform 2 (Fragment)  TOR (Fragment)  Heat shock protein family member  RPL9 protein-like protein (Fragment)  Beta-actin  Heat shock protein 90  Guanine nucleotide-binding protein (Fragment)  Rab5 (Fragment)  Anti-microbial Scy2  Ribosomal protein rpl13 (Fragment)  Ubiquitin carboxyl-terminal hydrolase  Endonuclease-reverse transcriptase-like protein  Catalase (EC 1.11.1.6)  ADP-ribosylation factor GTPase-activating protein  Clotting protein  Prohibitin (Fragment)  NADH-ubiquinone oxidoreductase chain 5 | 323.31  268.54  218.54  182.66  142.54  89.64  82.353  74.224  73.61  45.156  33.323  30.546  29.485  28.801  20.766  18.024  14.921  14.51  14.357  12.929  11.205  11.099  10.887  10.793  9.0336  7.2069  6.8863  6.6956  6.5571  5.9334  5.8973  5.8538  5.8235  5.7399  5.7269  5.6825  5.6281  -2 |
