# Supplementary material for: Exosomal miR-224 contributes to hemolymph microbiota homeostasis during bacterial infection in crustacean
Source: PLoS Pathog. 2021 Aug 11;17(8):e1009837. doi: 10.1371/journal.ppat.1009837 (PMC8382196; doi:10.1371/journal.ppat.1009837)
Supplement: Attachment — Submitted filename: Response to the decision letter.doc [file ppat.1009837.s004.doc]

**Response to the decision letter**

Editors

PLoS Pathogens

Date: Jul 13, 2021

Dear Editors,

We are pleased to know that our manuscript entitled “Exosomal miR-224 contributes to hemolymph microbiota homeostasis during bacterial infection in crustacean” (PPATHOGENS-D-21-00931) has the opportunity to be resubmitted. We would like to take this opportunity to appreciate your time and to thank the anonymous reviewers for their valuable suggestions and comments. The manuscript has been carefully revised and greatly improved for additional related experiments, data analysis and language writing, accordingly. All changes are highlighted with red color in the revised manuscript. The point-to-point answers to the comments of the reviewers are as follows.

**T****o Reviewer 1:**

***Part I - Summary***

Gong et al propose a manuscript entitled “Exosomal miR-224 contributes to hemolymph microbiota homeostasis during bacterial infection in crustacean”. The authors assess the role of extracellular vesicles released by V. parahaemolyticus infected mud crabs into the hemolymph. They claim that these extracellular vesicles have a protective role against Vp infection reducing crab mortality by maintaining the homeostasis of hemolymph microbiota. They focus on miR-244 which they found enriched in extracellular vesicles, and propose that it targets HSP70 downregulating its expression, which allows TRAF6 to interact with Ecsit ultimately regulating the expression of mROS and ALFs.

The data reported is relevant and of potential broad interest, in addition it may impact different fields of research. The manuscript has been previously submitted for publication in Plos Path. In this new version the authors assess the referee comments and performed additional experiments. However, the manuscript still lacks important details which perturb the proper appreciation of the data. This is a very extensive study but the authors fail to properly present the data and argument the findings. As an example, all the new performed experiments were added as supplementary data, with no effort to integrate some of them (which are of critical importance, as Fig S3) in the main manuscript. The new data and its implications are not exploited to improve the findings.

**Answer:** I agree with the reviewer’ s valuable comments, as suggested, the data of Fig. S2 and Fig. S3 have been move to Fig. 4 and Fig. 7, respectively. Besides, more detailed information has been provided accordingly in the revised manuscript.

**Revisions in the revised manuscript:** Fig. 4I; Fig. 7K; Lines 242-243; Line 323; Lines 778-781; Lines 855-857; Part II for details.

***Part II – Major Issues: Key Experiments Required for Acceptance Summary***

The authors need to look at all data and provide the controls missing, the quantifications, the details of the experiments and better images, and think about how they need to present them to convince the readers. The results section appears as an accumulation of observations and lack well supported conclusions, hypothesis, analysis in an integrative manner.

In general, the figure legends need to be more detailed: the kind of experiments should be mentioned, the number of animals, the number of independent experiments. The authors made the effort to add some of these in the material and methods section, but reduced info should also appear in Figure legends.

The quantification (all the quantifications) should be performed on at least 3 independent experiments and should appear as a graph including all the quantifications. Quantify the single experiment that is shown is not enough and only has a limited interest.

**Answer:** Based on the reviewer’ s detailed suggestions provided in Q4, Q5, Q7, Q8, Q10, Q12, Q13, Q14, Q15 Q16, Q17, Q18 and Q19 of Part II, these issues are addressed accordingly in the revised manuscript.

**Revisions in the revised manuscript****:** Part II for details.

**Abstract**

**Q1:** The abstract needs to be revised concerning the language and the content. For example, the first sentence is hard to follow. Actually, the Author summary is much clear than the scientific abstract itself. I strongly recommend revision.

**Answer:** Thanks for your valuable suggestions, as suggested, the abstract section is rewritten in the revised manuscript.

**Revisions in the revised manuscript:** The abstract section.

**Fig. 1**

**Q2:** Lines 157-159: The data does not demonstrate what is claimed. Instead, the data presented shows that extracellular vesicles derived from Vp-infected mud crabs have a protective effect on the mortality and proliferation of bacteria in the hemolymph, which may suggest a role in microbiota homeostasis and immune response.

**Answer:** As suggested, the relevant descriptions are modified accordingly in the revised manuscript.

**Revisions in the revised manuscript:** Lines 158-161.

**Q3:** A, B, C show that there is no major difference between Vp- or PBS-derived exosomes. The authors need to state this kind of conclusions.

**A****nswer:** As suggested, the relevant conclusion is added accordingly in the revised manuscript.

**Revisions in the revised manuscript:** Lines 138-139.

**Q4:** In the additional data presented in S1 there is no indication on the number of experiments.

**Answer:** Sorry for the carelessness, the experiments were performed in triplicates and the detailed information has been added to the figure legends of Fig. S1 in the revised manuscript.

**Revisions in the revised manuscript:** Figure legends of Fig. S1.

**Q5:** In F, why not counting the bacteria after plating. Counting under the microscope is a time-consuming way and is not as accurate. What fraction of the hemolymph was counted? From how many crabs?

**Answer:** Thanks for your kindly reminder, on this issue, we have compared plate counting and microscopic counting for crab hemolymph microbiota in our previous study, and found that microscopic counting method was much more suitable for crab hemolymph microbiota counting (Appl Environ Microbiol, 2018, 84, e02824-17. for details). Besides, in this experiment, each treatment was consisted of 5 crabs and there were three biological replicates for each group, meaning that each group represented 15 individuals. As suggested, more detailed information of hemolymph bacteria counting is provided in the relevant Materials and Methods section of the revised manuscript.

**Revisions in the revised manuscript:** Lines 502-510.

**Fig. 2**

**Q6:** Line 185: The data do not show definitively that exosomes modulate pathways that maintain the homeostasis of hemolymph microbiota by regulating the levels of mROS and ALFs…. This is a possibility. The statement needs to be down modulated.

**Answer:** As suggested, the statement has been modified accordingly in the revised manuscript.

**Revisions in the revised manuscript:** Lines 187-189.

**Q7:** Images in A are of low quality. They do not add something interesting.

**Answer:** As suggested, the ROS assay are reconducted and observed under high power microscope. All the relevant ROS images (including Fig. 2A, 3E, 5B, 6D) have been replaced with high quality pictures in the revised manuscript.

**Revisions in the revised manuscript:** Fig. 2A, 3E, 5B, 6D; Relevant figure legends.

**Q8:** In C, readers need to know the number of experiments and the number of crabs, this should be added to the legend.

**Answer:** Thanks for the suggestion, in Fig. 2C, each treatment contains 5 crabs and three independent experiments were performed. The detailed information is added to the figure legends of Fig. 2C in the revised manuscript.

**Revisions in the revised manuscript:** Lines 737-740.

**Q9:** In F, the authors should discuss why some species that are that are highly present (red) upon Vp infection, almost disappear in the presence of both Vp- and PBS-derived exosomes.

**Answer:** Thanks for your helpful advices, indeed, it was found that some bacteria highly presented during *V. parahaemolyticus* (*Lactobacillus*, *Moheibacter*, etc) were decreased in the presence of both exosome-Vp and exosome-PBS, we speculated that it might be mediated by the common bio-molecules packaged in them. As suggested, the point is discussed in the revised manuscript.

**Revisions in the revised manuscript:** Lines 184-187.

**Fig. 3**

**Q10:** In B, why do the authors analyse the expression of ALF1, given that in Fig2 they show that ALF4 is much more up-regulated? Data are presented relative to what? Housekeeping expression? What mimic-NC stands for?

**Answer:** Thanks for the comments. In Fig. 2C, we concluded that exosome-Vp could regulate the expressions of ALF1, ALF4 and ALF5, and they were all statistically significant. Thus, we chose ALF1 as the indicator for preliminary screening in Fig. 3B, and all the indicators were validated when potential miRNAs were identified (Fig. 3G). In view of the reviewers' concerns, we have revised the description to make it easier to understand.

Besides, data are presented relative to the value of Vp+ mimic-NC group, which are treated as standard “1”, and this could make it better compare the differences between them. mimic-NC stands for a disordered nucleic acid which serves as the negative control for the miRNA mimics. All of the above information has been added to the relevant figure legends in the revised manuscript.

**Revisions in the revised manuscript:** Lines 196-197; Lines 212-213; Lines 752-754.

**Q11:** It seems that some data presented in I, are the same that are shown in 2D. If this is the case, this should be clearly stated. Any other similar situations in the manuscript should also be stated.

**Answer:** Sorry for the confusion. In consideration for the cost of sequencing services, we did these sequencing experiments together when we designed, similarly, sequencing data of the same treatment group (Fig. 2D, 3I, 5F and 7J) only possess one copy and were uploaded to a public database. As suggested, the figure legends of Fig. 3I, Fig. 5F and Fig. 7J are modified in the revised manuscript.

**Revisions in the revised manuscript:** Lines 763-764; Lines 798-799; Lines 854-855.

**Fig. 4**

**Q12:** In D, data are presented relative to what???

**Answer:** This comment is similar to Q10, data are presented relative to the value of miR-224+ EGFP-HSP70-3’UTR group, which are treated as standard “1”. As suggested, the detailed information has been added to the relevant figure legends in the revised manuscript.

**Revisions in the revised manuscript:** Lines 772-773.

**Q13:** In E, F, G and H if quantifications shown as numbers in F and H are included in the graphs, the authors do not need to add them on the images of the gels… all those number are misleading!

**Answer:** It is a good suggestion. Based on the suggestion by reviewer and the presentation of similar experiments (Western blot) in *PLoS Pathogens* and other authorative journals (PLoS Pathogens, 2020, 16(5): e1008366; Cell, 2021, 184(3): 689-708), we added Gray-scale values to the pictures, instead of turning the Gray-scale values into the graphs. On the one hand, it can address the reviewer’s concerns to some extent, and at the same time, it can make our pictures consistent with *PLoS Pathogens* and other authorative journals. We hope our thoughts could be understood by reviewers and editors.

**Q14:** In I, the image needs to be accompanied by a quantification (through Pearson’s correlative coefficient or other that can measure co-localization). Data from the control added in this new version as supplementary should be added here, with corresponding quantification, of course. The readers need to know how much miR-224 colocalizes with HSP70 mRNA and with tubulin mRNA.

**Answer:** Thanks for the suggestions, and we have tried to do the Pearson analysis as suggested and found that the result was not satisfactory. After referring to similar studies, we learned that RNA Fish was different from protein immunofluorescence, and RNA distribution was diffused in cells, which was not suitable for this kind of analysis. Therefore, based on the suggestion by reviewer and the presentation of similar experiments (RNA Fish) in *PLoS Pathogens* and other authorative journals (PLoS Pathogens, 2020, 16(5): e1008366; Nature Communications, 2016, 7: 11215), we added arrows to indicate the colocalization in the pictures, which could make it more easy-understanding for the readers. In addition, as suggested by the reviewer, we move this part of the supplementary information (Fig. S2) to the main-text pictures. All the information mentioned above have been modified in the revised manuscript.

**Revisions in the revised manuscript:** Fig. 4I; Lines 242-243; Lines 778-781.

**Fig. 5 and 6**

**Q15:** Microscopy images showing mROS are of low quality.

**Answer:** As suggested, the cellular ROS assay in Fig. 5 and Fig. 6 (5B and 6D) and mROS assay in Fig. 7B are reconducted and observed under high power microscope. All the relevant ROS images have been replaced with high quality pictures in the revised manuscript.

**Revisions in the revised manuscript:** Fig. 5B, 6D, 7B; Relevant figure legends.

**Q16:** Western blots need to be quantified from at least 3 independent experiments and data shown as a graph, number do not need to appear on the images.

**Answer:** As suggested in Q13, we appreciate the reviewer’s kindly advices. Based on the suggestion by reviewer and the presentation of similar experiments (Western blot) in *PLoS Pathogens* and other authorative journals (PLoS Pathogens, 2020, 16(5): e1008366; Cell, 2021, 184(3): 689-708), we added Gray-scale values to the pictures, instead of turning the Gray-scale values into the graphs. On the one hand, it can address the reviewer’s concerns to some extent, and at the same time, it can make our pictures consistent with *PLoS Pathogens* and other authorative journals. We hope our thoughts could be understood by reviewers and editors.

**Q17:** Figure legends need to be carefully completed, as mentioned before. The important information and important details should be easily found.

**Answer:** Thanks for the suggestion, as suggested, the figure legends of Fig. 5 and Fig. 6 have been modified accordingly in the revised manuscript.

**Revisions in the revised manuscript:** Figure legends of Fig. 5 and Fig. 6.

**Fig. 7**

**Q18:** This figure should include data included in S3, this is a very nice piece of data that needs to exploited and shed to light in the manuscript.

**Answer:** It is a good suggestion, as suggested, the data in Fig. S3 has been moved to Fig. 7 as Fig.7K in the revised manuscript.

**Revisions in the revised manuscript:** Line 323; Lines 855-857.

**Q19:** Figure legend is poor, need to complete.

**Answer:** Thanks for the suggestion, as suggested, the figure legends of Fig. 7 have been modified accordingly in the revised manuscript.

**Revisions in the revised manuscript:** Figure legends of Fig. 7.

***Part III – Minor Issues: Editorial and Data Presentation Modifications***

The manuscript needs to be revised to correct language problems. A list of few examples is provided below, however there are much more along the text.

**Q20:** Line 31 “targeting to HSP70” needs correction. In addition, in this context, “target” is a vague term, the authors should be more precise may be “inhibit” or “block” or “downregulate the expression”.

**Answer:** As suggested, the sentence has been modified accordingly in the revised manuscript.

**Revisions in the revised manuscript:** Line 31.

**Q21:** Line 66 “are usually served as markers” needs correction.

**Answer:** As suggested, the description has been modified with “usually serve as indicators” in the revised manuscript.

**Revisions in the revised manuscript:** Line 66.

**Q22:** Lines 128-131 this sentence does not make much sense. May be a part of the sentence is lacking or a verb (“were isolated”??)

**Answer:** Thanks for the reviewer’s kindly reminder. Indeed, we left out a “were”, the sentence has been modified accordingly in the revised manuscript.

**Revisions in the revised manuscript:** Line 129.

**Q23:** Line 131-132 “then to evaluate the quality of the isolated exosomes, we performed a series of tests required” not very well written needs to be improved.

**Answer:** As suggested, the sentence has been modified in the revised manuscript.

**Revisions in the revised manuscript:** Lines 131-132.

**Q24:** Line 149 “were serves as control” needs to be corrected.

**Answer:** Thanks for the helpful advice. As suggested, we have modified the sentence with “served as control groups” in the revised manuscript.

**Revisions in the revised manuscript:** Line 150.

**Q25:** Line 154 “and therefore affecting the mud crab mortality” needs to be corrected.

**Answer:** As suggested, the sentence has been modified in the revised manuscript.

**Revisions in the revised manuscript:** Lines 153-155.

**Q26:** Line 212 “homeostasis of mud crab by exosomal miR-224 packaged” needs to be corrected.

**Answer:** It is a good suggestion. As suggested, the description has been modified accordingly in the revised manuscript.

**Revisions in the revised manuscript:** Lines 217-218.

**To Reviewer 2:**

***Part I - Summary***

The authors were able to address many of the concerns that I raised in the review comments. However, there are still several issues remaining to be addressed before the current manuscript is accepted for publication.

**Answer:** Thank you for the reviewer’ s approval of our previous revision, we hope that all the reviewer’s concerns can be solved in this revision.

***Part II – Major Issues: Key Experiments Required for Acceptance Summary***

**Q1:** The authors have attempted to address the questions of exosomal quality control that were raised during review. According to the definition of ISEV, exosomes are defined by the presence of two positive markers and a negative marker, such as GM-130, in addition to TEM and NTA. The authors should add MG-130 in the WB as a negative control (Fig. 1C).

**Answer:** I agree with the reviewer’ s comments, negative controls can help us to evaluate the purity of exosomes. In this paper, calnexin was used as the negative control, which has also been used in other similar studies (International Journal of Cancer, 2017, 141(3): 614-620). We have modified the relevant description in the revised manuscript to make it more easy-understanding. In addition, as suggested by the reviewer, MG-130 is also a good choice, but the species covered in this study is marine crustacean, and all the antibodies used are prepared in our lab, we will consider using this marker in our future studies if the antibodies is prepared.

**Revisions in the revised manuscript:** Lines 134-137; Lines 721-723.

**Q2:** In line 145, the authors stated that exosome-vp and exosome-PBS possess similar affinity to the recipient hemocytes. However, the Fig S1 shows the binding percentage not affinity of exosome-vp and exosome-PBS to hemocytes. You can’t measure affinity with a heterogenous population of exosomes for binding to cells. The authors should correct it.

**Answer:** Thanks for the helpful advices, as suggested, the relevant description for Fig. S1 has been modified accordingly in the revised manuscript.

**Revisions in the revised manuscript:** Line 146.

**Q3:** The authors acclaimed in multiple statements in the manuscript that miR-224 was upregulated or highly expressed in exosomal preparations without providing any descriptions on how the exosomes were and miR-224 were quantified. The authors should provide detailed description on how miR-224 was quantified in exosome-vp, such as if mass of exo-vp and exo-PBS (Fig. 3D) was used as a control or other exosomal markers were used as control.

**Answer:** Thanks for the reviewer’ s kindly reminder, we did miss this part of materials and methods. To quantify the levels of miR-224 in exosomes, U6 was used as internal reference in this study, which has also been used in other similar studies (Journal of Extracellular Vesicles, 2019, 8(1): 1629865; PLoS Pathogens, 2020, 16(5): e1008366). As suggested, more detailed information has been added to the Materials and Methods section of the revised manuscript.

**Revisions in the revised manuscript:** Lines 490-500; Lines 755-756.

**Q4:** The manuscript stated that exo-vp-miR-224 would accumulate in the recipient cells but did not present any evidence on this. The authors should either present evidence on the miR-224 accumulation or delete the statement.

**Answer:** Thanks for the comments, in Fig. 2D, we found that injection of exosome-Vp could led to the significant increased expression of miR-224 in mud crab hemocytes compared with exosome-PBS. Therefore, we concluded that exosome-Vp treatment could led to miR-224 accumulation in the recipient cells. As suggested, the relevant description has been modified in the revised manuscript to make it more easy-understanding.

**Revisions in the revised manuscript:** Lines 204-207.

***Part III – Minor Issues: Editorial and Data Presentation Modifications***

There are many other minor errors as indicated below but not limited to:

**Q5:** Line 54-55: “was further interacted with” should be “further interacted with”.

**Answer:** As suggested, the sentence has been modified accordingly in the revised manuscript.

**Revisions in the revised manuscript:** Line 32.

**Q6:** Line 66: remove “are” in “are usually served as”.

**Answer:** As suggested, the sentence has been modified accordingly in the revised manuscript.

**Revisions in the revised manuscript:** Line 66.

**Q7:** Line 76: remove “very” from “very little is…”.

**Answer:** As suggested, the sentence has been modified accordingly in the revised manuscript.

**Revisions in the revised manuscript:** Line 76.

**Q8:** Line 107: “uncontrol proliferation” should be “uncontrolled”.

**Answer:** As suggested, the sentence has been modified accordingly in the revised manuscript.

**Revisions in the revised manuscript:** Line 107.

**Q9:** Line 129: “exosomes isolated from” should be “exosomes were isolated from”.

**Answer:** As suggested, the sentence has been modified accordingly in the revised manuscript.

**Revisions in the revised manuscript:** Line 129.

**Q10:** Line 149: remove “were” from “were served”.

**Answer:** As suggested, the sentence has been modified accordingly in the revised manuscript.

**Revisions in the revised manuscript:** Lines 150-151.

**Q11:** Line 207: “A similarly trend” should be “A similar trend”.

**Answer:** As suggested, the sentence has been modified accordingly in the revised manuscript.

**Revisions in the revised manuscript:** Line 212.

**Q12:** Line 214-215: change “the target genes controlled by miR-224” into “the genes targeted by miR-224”.

**Answer:** As suggested, the sentence has been modified accordingly in the revised manuscript.

**Revisions in the revised manuscript:** Lines 220-221.

**Q13:** Line 216: change “the both two software” into “both software”.

**Answer:** As suggested, the sentence has been modified accordingly in the revised manuscript.

**Revisions in the revised manuscript:** Line 222.

**Q14:** Line 276: change “it was obviously” into “it was obvious”.

**Answer:** As suggested, the sentence has been modified accordingly in the revised manuscript.

**Revisions in the revised manuscript:** Line 282.

**Q15:** Line 294-295: change “in the ubiquitination of Ecsit inhibit” into “in the inhibition of Ecsit ubiquitination"

**Answer:** Thanks for the suggestion, “inhibit” is a typing error and has been removed. The sentence has been modified accordingly in the revised manuscript.

**Revisions in the revised manuscript:** Line 300.

I think that the revision would meet the reviewers’ comments and suggestions and our manuscript now is suitable for resubmission. In the case of any questions, please feel free to contact me.

Thank you very much!

Yours sincerely,

Shengkang Li

College of Sciences, Shantou University

China
